# A Qualitative Systematic Review of Older Persons' Perceptions of Health, Ill Health, and Their Community Health Care Needs

**DOI:** 10.1155/2013/672702

**Published:** 2013-05-07

**Authors:** Anne Lise Holm, Elisabeth Severinsson

**Affiliations:** The Centre for Women's, Family and Child Health, Faculty of Health Sciences, Vestfold University College, P.O. Box 2243, 3101 Tønsberg, Norway

## Abstract

The aim of this qualitative systematic review was to report a synthesis of older persons' perceptions of health, ill health, and their community health care needs. The review questions were what characterizes older persons' perceptions of health and ill health? and what are their community health care needs? Ten studies were identified in a systematic search for relevant qualitative papers published between January 2000 and January 2013 in the following electronic databases: PubMed, EBSCOhost/Academic Search Premier, and CINAHL. Publications were evaluated for quality, and a thematic analysis was performed. Two main themes were interpreted on a higher level: reconciliation with how life has become: and desire to regain their identity and sense of self-worth despite disability. Two themes emerged: creating meaning led to the experience of being valued in health care and society and a mental struggle to regain independence with the help of caregivers. Of special interest is the finding of perceptions related to the fear of becoming dependent on caregivers as well as the sorrow and pain caused by encountering caregivers who did not understand their desire to create meaning in their lives or their struggle for autonomy and independency.

## 1. Introduction

The concept of health has been characterised in many ways since the World Health Organisation (WHO) defined it as a state of physical, mental, and social well-being [1]. It is often described in holistic terms, including physical, psychological, social, cultural, and existential values [2–4]. The focus of the WHO [5] on increasing life expectancy has led to a marked growth in the older population globally, both in relative and absolute terms. This is true not only of high income countries but also of the rest of the world [5]. The increasing number of elderly persons will challenge global, national and local resources in the future [6]. The focus on active aging has made it a matter of increasing urgency to identify ways of maintaining elderly persons' health and well-being [7]. The health of such persons should not only be viewed in terms of disease prevalence or absence of illness but rather understood as two sides of the same coin. Older people's health is often associated with functional impairment, as physical functioning and psychosocial well-being are closely related [8–10]. Dependency on care has been negatively associated with health among older people [11–13]. Strandberg et al. [13] revealed that dependency on care is a struggle against worthlessness, powerlessness, loneliness, and failure to obtain help. According to Saveman [14], dependency can lead to a negative balance of power from the older person's perspective. McDonald-Miszczak et al. [15] stated that elderly persons' health appears to consist of two components: objective assessment and subjective belief. Cross-cultural differences in the effect of religion on health were found in the study by Gesler et al. [16]. Diener [17] stated that subjective well-being includes happiness, life satisfaction, and positive effect. For decades, the literature largely ignored positive subjective well-being, although human unhappiness was explored in depth [17]. Some years ago Campbell [18] stated that older persons reported greater life satisfaction except in the area of health. Some qualitative research has outlined caregivers' perceptions of what older persons need as well as exploring aspects of healthy aging [19–21], ill health and illnesses [22, 23]. 

Many who suffer from chronic ill health struggle with physical, psychological and social problems without help or support from the health care system [24]. Moreover, the help available fails to provide optimal clinical care or meet their needs to effectively manage their ill health [24]. Thus, community health care should develop nursing strategies in order to address the needs of elderly persons [5]. The World Bank predicts that by 2050, at least 25% of the public expenditure of developed nations will be allocated to health care, and pensions, leading to the risk of a crisis [25]. Community health care comprises the following: delivery of health care services including primary and secondary prevention, treatment, care and rehabilitation; activities that enable the delivery of health services, especially finance and resource generation; and stewardship functions aimed at influencing the health impact of interventions in other sectors, irrespective of whether or not their primary purpose is to improve health [26], [27, page 5]. 

Some theoretical and quantitative research has revealed important aspects of ageing. Berkman and Glymour [28] discussed the role of society in addressing the consequences of old age. Undeniably, everyone will face these difficulties as part of the aging process, which is influenced by social interaction, inclusion, economic status, education level and vulnerability to stress. The survey by Elo et al. [29] revealed that in order to support older persons to continue living at home, professionals must identify each individual's own perceptions about her/his health and complex health care needs. Interventions for older persons need to integrate knowledge about useful, safe, and appropriate changes and help them to acquire such strategies [30]. 

The rationale for this review was to gather qualitative knowledge of older persons' perceptions of health and ill health in order to better understand their community health care needs. Do older persons consider ill health the opposite of health and an inevitable part of the ageing process? Do they perceive themselves as healthy despite ill health? The authors were unable to find a qualitative systematic review study that focused on older persons' perceptions of health, ill health, and community health care needs. 

 The aim of this qualitative systematic review was to report a synthesis of older persons' perceptions of health, ill health and community health care needs. The review questions were: what characterizes older persons' perceptions of health and ill health? and what are their community health care needs?

## 2. Methods 

The systematic review method was used to gather existing qualitative knowledge [31]. A strong case has been made for the potential of qualitative research to inform policy and practice [32, 33]. There seems to be no consensus on appropriate guidelines for the systematic review of qualitative evidence in the areas of health and social care, and the whole process of synthesising qualitative research is hotly debated [34, 35].

### 2.1. Literature Search

**Figure 1:**
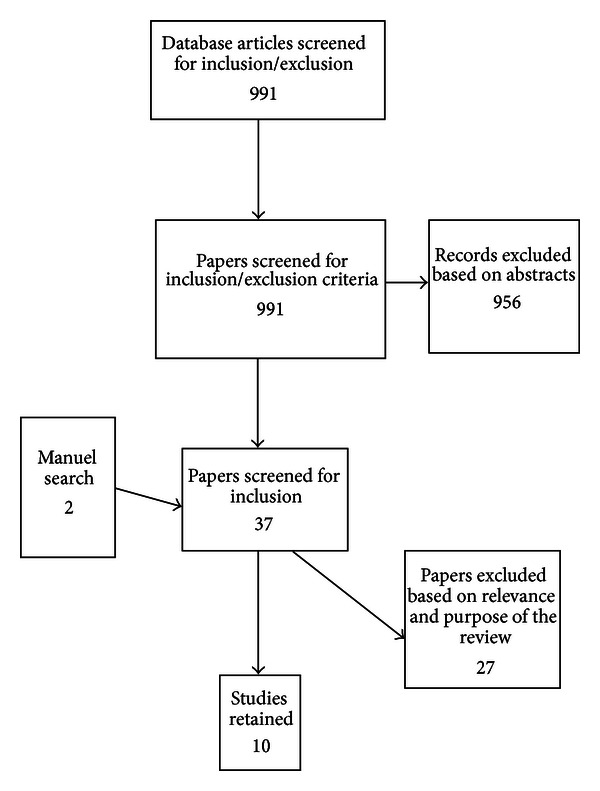
Search and retrieval process.

The studies included in this review were identified by a systematic search for relevant papers published between January 2000 and January 2013 in the PubMed (410), EBSCOhost/Academic Search Premier (369), and CINAHL (212) electronic databases. The following search words were used in combination and separately: old, older, elderly, health, health care, ill health, illness, nursing, qualitative (Figure 1).

### 2.2. Inclusion and Exclusion Criteria

The inclusion criteria were older persons over 60 years of age living in the community, qualitative studies published in English, empirical research, narratives, and experiences of health and ill health. The exclusion criteria were resident in nursing homes, professionals' experiences or perceptions, and review studies.

### 2.3. Thematic Synthesis

According to Thomas et al. [36], thematic synthesis draws on primary qualitative research and other established methods. A thematic synthesis can identify a range of common themes as well as any divergent views [34] and seeks to expand understanding of a phenomenon, patient experience, or perception [35]. The degree and type of interpretation can be a major issue for a reviewer attempting to synthesise data from several different primary studies. 

**Figure 2:**
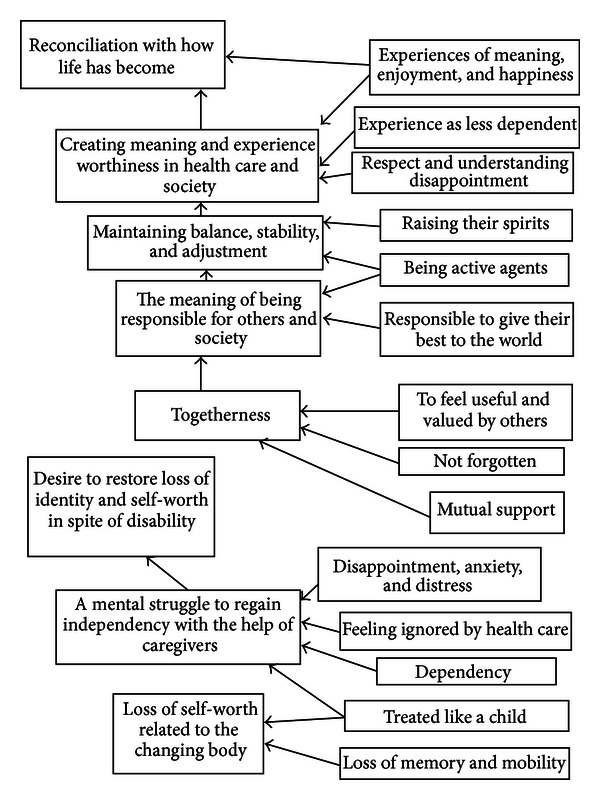
The themes and sub-themes that emerged from the descriptive themes.

The authors organised and abstracted the findings in accordance with the themes identified in the data. In this process, they applied the stages described by Thomas et al. [36] and Thomas and Harden [34] for developing a thematic synthesis. In the first stage the authors read the studies carefully to gain an overall impression of perceptions of health, ill health, and community health care needs, using free line-by-line coding. Each statement was associated with the older persons' perceptions of health, ill health, and their community health care needs. In the second stage, pen and paper were used to develop the codes into descriptive themes (Figure 2). In the final stage, the authors used the descriptive themes in the interpretation of a new thematic synthesis that went beyond the original studies (Table 2). According to Thomas et al. [36], the synthesis identifies groups and summarises the findings of the included studies. The term “thematic synthesis” has been described as a synthesis research method, although its steps are unclear, and few examples were found of synthesising qualitative data where the themes emerged from the analysis process [37]. The thematic synthesis comprised an independent review of the studies by the two authors, during which they discussed the themes to ensure that they reflected the included studies, after which they scrutinised the text to establish whether or not other themes emerged. The interpretation of the themes was important for reaching consensus and can be related to Lincoln and Guba's [38] concept of credibility. This sorting and labelling process concerned searching for the underlying meanings embedded in the included studies. The emerging themes were reexamined to ensure trustworthiness [39]. 

### 2.4. Quality Assessment

There is little consensus regarding how or whether quality can or should be assessed at all in relation to qualitative research [34]. The authors of this review assessed the impact of study quality on the findings in accordance with the 12 criteria suggested by Thomas and Harden [34]. Five criteria relate to the quality of the reporting of a study's aims, context, rationale, methods, and findings (were the sample, selection, and recruitment methods adequately described?) [34]. Four criteria relate to the strategies employed in the methodological assessment. The methodological criteria recommended by Lincoln and Guba [38] and Polit and Beck [31], especially the concept of trustworthiness, were used to discuss the included studies. The final criterion relates to the assessment of the appropriateness of the study methods for ensuring that the findings about older persons' perceptions of health and ill health were rooted in their narratives (e.g., were the data collection methods suitable for facilitating older persons to express their perceptions?). We also ascertained whether a description of the steps or stages in the analysis process was provided including the type of analysis performed (see Table 1) in addition to assessing demographic characteristics and ethical aspects (approval) of the selected studies. 

## 3. Results 

The search revealed 991 abstracts, in addition to review, qualitative, quantitative, and theoretical papers, of which the vast majority did not meet the inclusion criteria. A subsequent manual search related to the content of the relevant papers and their significant references revealed two additional studies, which were assessed for inclusion. In total, 37 empirical papers were retrieved and read through, after which ten qualitative studies were included. 

### 3.1. Aims

Although the aims of the included studies differed, some common aspects were deemed adequate for the purpose of this qualitative systematic review. The aim of three studies was to obtain a deeper understanding of older persons' views, perspectives, and lived experiences associated with health and well-being [40], health empowerment [41], as well as health and its meaning and significance [42]. Five studies explored older persons' problem solving [43], coping with daily life [44], ethos, and factors that influence access to health care [45], healthy ageing, and perceived influences on healthy ageing [46], health resources and strategies [47]. One study analysed the views and experiences of older persons from rural areas [48], while another described the daily lived experiences of older persons with chronic health problems [49].

### 3.2. Health Service Context and Recruitment of Participants

The information about context and recruitment varied. From et al. [40] study was conducted in three Swedish communities and the participants' needs were identified by a professional care needs assessor in the respective community. The study by Crawford Shearer [41] lacked information about the context but stated that the participants were recruited through the community meal-delivery programme. van Maanen's [42] sample consisted of older white, American, and British-born participants recruited through community centres, personal contacts, a university and local health authorities [42]. H. H. Tsai and Y. F. Tsai's [42] study was conducted in the Hsin-Cheng district of Hualien county in eastern Taiwan, and the participants were recruited by public health nurses and fellow participants. Birkeland and Natvig's [44] study was carried out in two municipalities in three home nursing care districts in Norway. Ten participants were recruited by a day-care centre [44]. Bentley's [45] study took place in a village in England and the participants, who were members of the social club (a local meeting place), were recruited by a physician. Tohit et al.'s [46] study was part of a larger cross-national study in Australia, China, and Malaysia, and the participants were recruited by a community leader known to the first author. Kulla et al.'s [47] study formed part of a project to introduce homecare visits among older Swedish-speaking Finns, but no information about recruitment was reported. Manthorpe et al.'s [48] study context was rural communities in England. A research team recruited elderly people and their carers within community organizations [48]. Jacelon's [49] study from central New England lacked information about the participants' needs. 

### 3.3. Design and Data Analysis

The design and analysis method used in the included studies are presented in Table 1. The steps of the data analysis were presented in various ways. All studies described how themes or categories emerged from the data.

### 3.4. Demographic Characteristics

The total sample in the included studies comprised 331 participants with a mean age of 74 years, 97 of whom lived alone. Five studies provided more detailed demographic characteristics such as education level [41, 43, 46], marital status [41, 43, 45, 46, 49], number of years in village [45], transport [40, 45], and activities of daily living (ADL) [43] and income [41].

### 3.5. Ethical Approval

Seven studies approved by an institutional review board [40, 41, 43, 44, 46, 47, 49] stated that research ethics were considered, and autonomy, confidentiality, and anonymity were guaranteed. Manthorpe et al.'s [48] study was part of a project for which formal ethical approval was not required. 

### 3.6. Methodological Assessment

In order to ensure trustworthiness, the authors attempted to avoid bias by not focusing on one study at the expense of another, as recommended by Pope et al. [50]. Lincoln and Guba [38] described trustworthiness in qualitative research using the concepts of credibility, dependability, confirmability, and transferability. Two studies contained information about how to enhance trustworthiness [43, 49]. H. H. Tsai and Y. F. Tsai [43] and three other studies used the concept of credibility [40, 42, 45]. Birkeland and Natvig [44] stated that “*conformability*” concerns the selection of informants and how they express and interpret their experiences. These authors may have meant “confirmability”, which has the same meaning as objectivity and is the degree to which study results are derived from the characteristics of the participants and context and not influenced by researcher bias as outlined by Polit and Beck [31]. Two studies employed member checking to validate what was communicated during the interview [43, 49]. Schneider et al. [51] who used the peer checking method considered member and peer checking techniques important for establishing the creditability of qualitative date. Two studies [46, 47] did not use the concepts of credibility, dependability, confirmability, or transformability. 

In the review process it was important to provide feedback about the emerging themes and interpretations to ensure agreement among the two authors about the representativeness of the included studies. The present review is secondary research that involves a study conducted by someone other than the original researcher [31]. Credibility refers to confidence in the truth of both the data and their interpretation [31]. The fact that the included studies have different aims makes it challenging to conduct a trustworthy analysis. Confirmability should be derived from the characteristics of the participants (here the included studies) and context and not the authors' preunderstanding [31]. 

Reviews are secondary research prepared by someone other than the original researcher [31]. A thematic synthesis is related to credibility, which concerns confidence in the interpretations and enables validation of the data [31]. As the number of published studies is increasing, the search strategy can be either too broad or too narrow and new evidence could change the relevance of a review in terms of the concept of dependability, that is, the stability of data and conditions over time. Thus the possibility of excluding relevant studies is ever present. The results of a systematic review cannot be directly transferred to experiences of health, ill health and health care service in other parts of the world. The readers are responsible for concluding whether or not the results of a review are applicable in their own context, as explained by Lincoln and Guba [38]. Five studies were from Europe (two from the UK and three from the Nordic countries), two from the USA, one from Canada, one from Australia but only one from the eastern hemisphere (Taiwan). The culture reflected in the studies may vary between west and east. The different experiences of health, and ill health all over the world must be taken into account. Further studies in other countries are recommended in order to strengthen the trustworthiness of qualitative research. 

### 3.7. Assessment of Appropriateness

As recommended by Thomas and Harden [34], the study methods must ensure that the findings are rooted in the perceptions of the older persons. Thus the aim, design, and analysis used in the included studies were assessed in terms of their appropriateness for helping the older persons to express their perceptions and views. 

### 3.8. The Result of the Thematic Synthesis

Two main themes were interpreted on a higher level: reconciliation with how life has become and desire to regain their identity and sense of self-worth despite disability. Two themes emerged: creating meaning led to the experience of being valued in health care and society and a mental struggle to regain independence with the help of caregivers. the first theme was based on three subthemes: maintaining balance, stability, and adjusting, the meaning of being responsible for others and society, and togetherness. The second theme comprised one subtheme: loss of self-worth related to the changing body.

#### 3.8.1. Reconciliation with How Life Has Become

Health can be interpreted as reconciliation with how life has become and associated with personal characteristics. A positive attitude to life can be part of reconciling oneself to difficult aspects of ill health. However, reconciliation does not involve resignation but rather acceptance of one's fate or situation. A term used by van Maanen [42] is “destiny”, defined as being in charge of one's life, having a future to look forward to and perspectives that make life worth living. One of the participants said:

*“I have a bird that sings. I am glad to be alive and feel that the bird is singing within me. The bird gives me a feeling of joy, happiness at being alive”* [42, page 56].

Positive attitudes were also important for reconciliation. Spirituality and faith seemed to help the participants to retain a positive attitude [49]. When asked why she went to Mass, one woman said:

* “Well, I feel better when I go. I speak to God for a little while”* [49, page 19].

*(1) Creating Meaning Led to the Experience of Being Valued In Health Care and Society*. The positive attitude to life was related to creating meaning, which led to the experience of being valued in health care and society [40, 48]. From et al. [40] stated that feeling healthy was connected to experiences of meaning, enjoyment, and happiness in life. These authors reported that taking part in meaningful activities in society indicated that elderly persons were not ignored, which made them feel respected [40]. One woman commented

* “I do not feel bad and sit here crying because I'm alone. No, no. I'm not as dependent as others. I feel sorry for those who must have people around them”* [40, page 283].

Manthorpe et al. [48] revealed that older people need to be valued and have their complaints taken seriously, although they must learn to accept the fact that changes are an important aspect of life. Jacelon [49] reported that the most physically impaired and chronically ill participants were the most positive. Strategies to foster an affirmative attitude included maintaining a positive outlook, finding and creating meaning in one's life, accepting losses, and planning for the future.

*(a) Maintaining Balance, Stability, and Adjusting. *Maintaining balance, stability, and adjusting [40, 41, 43, 44, 46, 47, 49] was described as a way to regain health. From et al. [40] explained that the participants' ability to feel healthy depended on their own capacity for adjustment and compensation. Being encountered with respect and understanding made it easier to adjust and accept the compensatory activities provided by their care givers. Mutual concern on the part of both the caregiver and the older person added to the experience of well-being [40]. The participants in the study by Crawford Shearer [41] spoke of the physical changes that limited them and kept them in their homes. H. H. Tsai and Y. F. Tsai [43] demonstrated that elderly persons' stories reflect a struggle in which they tried to accept their fate and avoid disappointment. Unwillingness to seek help may indicate a desire to save face, which is common in Chinese culture [43]. One of the participants who had adjusted to living alone for many years said

*“When I'm not in a good mood I put up with it. What else can I do? This is my fate”* [43, page 984].

Birkeland and Natvig [44] emphasised that acceptance is not the same as giving up, but the elderly person has to struggle to go on, which becomes a part of her/his life philosophy. Tohit et al. [46] emphasised that having enough money was important for satisfying material needs but that spiritual endeavour balanced material desires and assisted the participants to live a more peaceful life. Kulla et al. [47] stated that adjustment means more flexibility in coping that allows elderly persons to perceive themselves as less dependent. Health varied between good and poor when age was taken into account [47]. In the study by Jacelon [49], the participants explained that they had to accept and be content with life and that their attitudes were important for maintaining balance. The older persons continuously refined their balance by making adjustments between activity, attitude, autonomy, health, and relationships [49]. 

*(b) The Meaning of Being Responsible for Others and Society.* Existential dimensions of life were revealed in four studies that described the need to be responsible for others and society [45, 47–49] in order to ensure health and longevity [45]. Bentley [45] revealed that belonging and contributing to village life as well as coping with health problems themselves were traditional ways of maintaining health. Kulla et al. [47] reported that although elderly persons did not exercise their consumer rights, they believed that they had the same rights as younger persons in the allocation of health resources. The participants stated that being active meant developing strategies “to loosen up”, which raised their spirits and enhanced their zest for life [47]. Manthorpe et al. [48] described the participants' concern about changes in the health care infrastructure and services that led to the perception that these services were gradually moving beyond their reach. The centralization of health services made it difficult to attend appointments. One important aspect was the need to emphasize that the older persons were responsible and active agents in their own constructions of well-being in the community [48]. Jacelon [49] reported that some of the participants found meaning in being responsible for others and society. Meaning was derived from engaging in day-to-day activities, in some cases as an expression of their faith, while others wanted to leave a legacy [49]. One participant talked about his duty to give his best to the world and stated

*“I once heard a poem, it was long, but the final words were: “Give the world the best you can…and it still may not be enough. Give the world the best you can anyway!”* [49, page 19].

*(c) Togetherness.* Another aspect of reflections on life and a positive attitude emerged in seven studies that outlined the meaning of togetherness [40–42, 44, 47–49]. From et al. [40] described togetherness as social support, relationships, being involved, contact, closeness, trust, hope, and being valued, respected and not forgotten by others. Crawford Shearer [41] stated that members of the lung disease study group became friends who turned to each other when they had questions about living with the disease. van Maanen [42] reported that social exchanges such as sending small gifts that could not be left unacknowledged were used by the participants as a means to strengthen and renew relationships with relatives and friends. Such strategies were described as a way to remain involved and visible. The same strategies were employed to some extent in relation to nursing staff. When the older persons' physical needs were met, they rewarded the nurses with gifts of chocolates, praise, and so forth [42]. Birkeland and Natvig [44] found that even in cases where the elderly person was alone for most of the time, she/he did not necessarily feel lonely if perceiving the contact with family and friends as good. Kulla et al. [47] reported that social support and relationships became more important when the elderly persons perceived them as an opportunity to feel useful. Loneliness was experienced when dealing with the subject of death, although the elderly persons did not feel alone when their closest relatives were nearby [47]. Manthorpe et al. [48] mentioned that the benefits of growing old in the countryside were related to the sociability and mutual support of persons in the local communities. Elderly persons' relationships were complex, as they had less friends and experienced occasional loneliness. One of the participants stated

*“Where I live there are lots of elderly people. We all help each other. We're very independent. The area is generally good for people with disabilities—in villages and small towns such people are accepted and included—maybe less so than previously but it is still pretty good*” [48, page 465].

Jacelon [49] described that reciprocity was important in most relationships. The strategies used to maintain relationships included engaging in social situations and negotiating family roles. 

#### 3.8.2. Desire to Regain Their Identity and Sense of Self-Worth Despite Disability

Ill health was interpreted as a desire to regain their identity and sense of self-worth despite disability associated with a loss of functions related to physical, mental, and social dimensions, as most of the participants in the included studies had a chronic disability and thus impaired functional capacity. Such loss appeared to cause disappointment, anxiety, and distress, especially if adequate help was not available. Thus the desire to regain their former identity can be related to functional disability on several levels. Dependency on community health care was an endless struggle to gain autonomy and independence. Their zest for life appeared to decrease, leading to a feeling of despair. Negative emotions seemed to give rise to further disability and influence the older persons' relationships with family and friends. van Maanen [42] explained that the participants' self-image was distorted by health problems, which resulted in overemphasis of their own physical and mental functioning and rehabilitation. Health professionals became an extension of the self; the “we” were regarded as allies and acted as exponents of healing in a life crisis [42]. 

*(1) A Mental Struggle to Regain Independence with the Help of Caregivers.* Five studies described ill health as involving a mental struggle to regain autonomy and independence, in which feelings of despair and anxiety dominated daily life [40, 42, 46, 47, 49]. From et al. [40] reported that lack of independence meant being unable to do the things they were accustomed to doing and being dependent on others for tasks they had managed themselves throughout their lives. This was related to the caregivers' attitude as well as ability to identify and respect the older persons' desires, needs, and problems [40]. van Maanen [42] explained that impairment and rehabilitation constituted a process from complete dependence towards gradual independence, with the knowledge that in some cases complete independence would probably never be achieved. The statements reflected the participants' concern and anxiety about the future, which influenced their experiences of ill health [42]. Tohit et al. [46] described that being physically independent was important for the participants, especially the ability to carry out their religious activities. Some considered themselves ignored by the health care system or felt that they were treated like a child and shown no respect because of their age [47]. Jacelon [49] reported that the participants received most of their healthcare information from their provider but made their own decisions about how to use it. Many designed a health management plan for themselves that included interventions prescribed by health care providers as well as personal strategies, including monitoring their health status, keeping track of medications, and learning to live with the disease [49]. A participant with increasing blindness stated

*“Self-reliance is important. I have to keep trying to do things myself so I won't become dependent”. It's a desire not to be helped when I do not think I need it—I tell them that when I really need help I'll ask for it”* [49, page 20].

*(a) Loss of Self-Worth Related to the Changing Body. *Ill health was also described as sorrow, pain, and loss of self-worth related to the changing body. Ill health was older persons' way of coping with illness [40, 41]. From et al. [40] described ill health as the opposite of the idealistic view of health. It meant being unable to live as one would like loss of memory, immobility, and pain. Such experiences were associated with sadness, anxiety, and sorrow. The latter was often related to losses such as physical abilities, loved ones, and/or a private life [40]. Crawford Shearer [41] reported that the participants spoke of physical changes that limited and kept them in their homes. They acknowledged their need for oxygen, wheelchairs, and other life-sustaining devices [41]. In relation to her hands, one woman said: “I wonder what they'll do when I cannot sign my name any more?” [41, page 24].

## 4. Discussion 

The aim of this qualitative systematic review was to report a synthesis of older persons' perceptions of health, ill health and their community health care needs. The review questions were what characterizes older persons' perceptions of health and ill health? and what are their community health care needs? 

Two main themes were interpreted on a higher level as characteristic of the perception of health, ill health, and community health care needs; reconciliation with how life has become and desire to regain their identity and sense of self-worth despite disability, Two themes emerged: Creating meaning led to the experience of being valued in health care and society and a mental struggle to regain independence with the help of caregivers. Health seemed to be experienced as *reconciliation with how life has become. *A positive attitude to life was important and nursing care could help remove some of the obstacles. Despite their disabilities, the older participants appeared to struggle to feel healthy [47]. Health was not something they consciously worried about and they did not become obsessed with their physical and mental status. According to Gadamer [52], health is part of the wonder of being able to forget oneself. It can be a function of lifestyle and emotions, where social life, physical and mental health are components of well-being. Aging appeared to be considered beyond personal control, which can indicate resignation to a situation one is unable to change. Thus in order to live a healthy life, the older person needs help to overcome resignation. Erikson's [53] theory describes a final perspective on life, where the task is to develop an attitude of retreat and retirement from the world. This eighth stage has been characterised as an attempt to achieve spiritual reconciliation at the end of life [54] and involves a shift from a materialistic view of the world to a sacred dimension. If reconciliation is not achieved, one can experience emotional pain [55] or a feeling of estrangement [56].

*The experience of being respected and valued by the health care system and society *seemed to raise the older persons' spirits. The need to create meaning and feel valued seems deeply rooted in human nature [57–59]. Understanding the life of an older person in a holistic way in order to see her/him as valuable and worthy of respect seems important. Greater awareness of the meaning of participation is necessary in community health care to avoid violating older persons. The perception of being incapable can lead to imbalance. Health care professionals are sometimes unaware of the necessity to demonstrate commitment and create trust. The lack of commitment can be related to negative attitudes about ageing and older people, for example, their inability to be in charge of their life. As highlighted by Manthorpe et al. [48], the community health care system must cater for minority groups of older persons, some of whom seem to have diminishing trust in the Western health care system. Such distrust can be related to the fact that nurses fail to ask how an elderly person with a different cultural background experiences her/his health and ill health, which may be due to fear caused by personal prejudices [52]. Health care professionals require a culture characterised by open mindedness. However, the acquisition of cultural competencies takes time, after which they must be built into the system. The consequence of an absence of such competencies can be that nurses and other professionals lack knowledge of how to include and help older persons with a different cultural background. Facilitating discussion of and reflection on their own attitudes and emotions can enable nurses and other health care professionals to define health and ill health, develop sensitivity to older persons' loss and suffering, help them to process their feelings and emotions. Assessment of elderly persons' previous ways of creating meaning can provide insight into their health beliefs and patterns. 

As older persons have been described as *maintaining balance, stability, and adjusting*, an awareness on the part of nurses and health care professionals of older persons' perceptions, health care requirements, and the fact that they need to feel accepted can empower these individuals by strengthening their sense of control and identity. The need for acceptance may be related to the view that older persons are less valuable than younger ones. Unwillingness to seek help seems to reflect a desire to save face as described by H. H. Tsai and Y. F. Tsai [43]. In most cultures saving face represents a way of avoiding shame and can help to maintain acceptance, dignity, prestige, and/or reputation. Older people do not want to make too many demands on others and merely desire a constructive dialogue about their health. Gadamer [52] explained that health can be seen as a balance and mystery, where body and soul work together to achieve harmony. Such harmony underlies healing and is the secret of health [52]. Thus, health and well-being can be related to feeling safe and secure, which has important implications for nurses and other health care professionals. 

*The meaning of being responsible for others and society* can be related to recent changes in community health care that focus on the *personal responsibility to be active* and is the reason why older persons have protested to the authorities. However, their protest seems to have had no effect [48], resulting in a sense of being excluded from society. Inclusion, confirmation, and trust appeared important for preventing older persons from becoming passive, alienated and developing a sense of not belonging. Physical changes as a consequence of the ageing process require nurses and other health care professionals to help older persons adapt to new social, political, and economic structures. Health can be related to elderly persons remaining active in their own community [48]. Dependence on health care sometimes results in a feeling of helplessness that makes it difficult to be in charge of one's life, that is, self-management. Maintaining health can be considered both a personal and a community responsibility as well as a form of self-affirmation [60]. Health maintenance might be explained by how societies define ageing and health [61]. 

*Togetherness *can be related to the positive influence of social support and relationships on the health of older persons and can serve as a recovery strategy. The nurse manager and health care provider have an obligation to identify groups of older persons at risk of developing ill health, such as those who are widowed, ill, or isolated and lack social contact and support [62]. Resilience is dependent on building and maintaining relationships that can help to strengthen self-worth and seems to be an unending developmental circle [53]. In the case of older persons living alone in rural communities or who have no family nearby, the team can play a role in arranging meetings where older persons can build a new social support system and help each other.

*Desire to regain their identity and sense of self-worth despite disability *can be related to decreased self-worth and health. Receiving help from family members and being dependent on care can be associated with a bitter feeling of dependency, helplessness, and powerlessness. As the results of this review revealed, the older persons' ill health seemed to involve *a mental struggle to regain independence with the help of caregivers.* Today's health care services are medically dominated, which emphasises the power of health care professionals and the powerlessness of older persons [24, 45]. All health care professionals, including physicians, need to become aware of the necessity to treat the older person as a valuable partner who is an expert on her/his health status and to adopt a way of thinking that empowers patients. If older persons are not allowed to make their own decisions, they can slip into a passive role because their opinions are considered irrelevant and they have no power, which makes them feel rejected and misunderstood. Thus health care professionals should promote positive attitudes that can influence the older person's self-worth. In such cases it may be important to be aware that when feeling unworthy and powerless, human beings have a low expectation of involvement and little interest in making their voices heard [45]. 

*Loss of self-worth related to the changing body* has several implications for nursing practice and health care services. One of the most challenges seems to be helping the older persons to develop their physical and mental capacity, thereby strengthening their self-worth and ability to express the sorrow and pain related to loss of function. Health care professionals, especially nurses, have a duty to identify and respect the older persons' physical and mental wishes and needs related to the changing body [24, 62]. Ill health can imply a lack of self-management, a sense of estrangement and a loss of togetherness [62], in which state the older person has no strength to fight for her/his rights. From et al. [40] stated that the opposite of togetherness is being an onlooker, which meant loneliness, lack of close relationships, and being excluded and ignored. 

### 4.1. Conclusion

In conclusion, the importance of community health care is clearly evident in the findings. Of special interest are the perceptions related to the fear of being dependent on caregivers and the sorrow and pain experienced by older persons when caregivers do not understand their desire to create meaning in their lives or their struggle to regain autonomy.

## Figures and Tables

**Table 1:** Studies included in the qualitative systematic review of health and ill health among old persons' in need of health care.

Authors, year, country	Design, analysis	Sample	Summary of the outcome
(1) Bentley (2003) UK [45]	Ethnographic design. Observations and interviews. Interview questions focused on the use of primary care services. Ethnographic analysis.	*N* = 9 (6 women, 3 men). 2 lived alone Community dwelling English older adults. Age ranged from 68–86 years of age.	Cultural factors were found to influence coping in health and illness and to legitimise primary health care access. No informant found it necessary to exercise her/his rights as a health care consumer, suggesting that despite initiatives to involve patients as partners in health care, the hierarchical position of the elderly people in the village remained unchanged since the days of the medical model and constituted a significant barrier to their use of health services.

(2) Birkeland and Natvig (2009) NORWAY [44]	Hermeneutic design. Hermeneutic analysis.	*N* = 20 (12 women, 8 men) All lived alone Community dwelling Norwegian older adults Age ranged from 72–93 years All the participants reported living with different chronic illnesses and extensive dysfunction.	The findings revealed that even when physical constraints limited their level of activity, the elderly persons were able to adapt and carry out various activities that did not require physical strength. The main coping strategy comprised accepting the situation, which often took the form of a resigned and passive attitude.

(3) Crawford Shearer (2008) USA [41]	Phenomenological design, Phenomenological analysis.	*N* = 14 (women) Seven lived alone Twelve white, one Hispanic, and one native American community dwelling older adults Age from 69–94 years The women suffered from different chronic illnesses.	The theme of embodiment emerged with theme clusters of caring for the body, viewing their body, and acknowledging changes that explain the lived experience of a changing self and environment, particularly the role of such changes in health empowerment. A description emerged of the self as changed by aging and chronic illness; this description wove together meanings of the past, present, and future.

(4) From et al. (2007) SWEDEN [40]	Inductive design. Individual interviews. Content analysis.	*N* = 19 (12 women, 7 men) 16 lived alone 12 received help both day and night. Community dwelling Swedish older adults. Age from 70–94 years. 15 suffered from different illnesses of whom 12 received help day and night.	The findings suggested that the possibility to feel healthy was dependent both on the older person's ability to adjust or compensate to their situation and on how their caregivers, relatives, and friends could compensate for the obstacles the older person faced. The subcategories that captured the informants' experiences of health and ill health were described as positive and negative poles of autonomy, togetherness, tranquility, and security in daily life. The significance of the caregivers was clearly evident. Their competence, commitment, and treatment were prerequisites for the older person's ability to experience health in spite of being dependent on care.

(5) Jacelon (2010) USA [49]	A qualitative descriptive design. Individual interviews. Constant comparative analysis.	*N* = 10 (6 women, 4 men) Six white, two Afro-American, two immigrants, one Hispanic and one from Ireland. They were community dwelling adults. Some lived alone, others with spouses or children. Age from 75–98 years All the participants had a range of chronic health problems and illnesses.	The participants' health problems varied and they developed strategies to maintain balance by means of activity, attitude, autonomy, health, and relationships. This research revealed a new perspective on living with chronic illness, and the model might provide a framework for rehabilitation nurses who work with older adults.

(6) Kulla et al. (2006) FINLAND [47]	Hermeneutic approach. Hermeneutic analysis.	*N* = 22 (13 women, 9 men). Community dwelling Swedish-speaking Finns. Age 75 and over.	The main health resources and strategies employed by the elderly Swedish-speaking Finns were related to social and other activities as well as to personality. Transforming health obstacles into resources could be an important health-promoting nursing strategy.

(7) Manthorpe et al. (2008) UK [48]	Mixed methods design. Inductive analyses.	*N* = 120 Community dwelling adults. Data collection was not described.	Three overarching themes underpin elderly persons' views on health and well-being in rural areas: the changing characteristics of rural communities, the relocation and reconfiguration of health and social care services, and the balance between positive and negative aspects of rural life.

(8) Tohit et al. (2012) AUSTRALIA [46]	This study is part of a larger cross-national study. Focus groups.	*N* = 38 (20 women, 18 men) Community dwelling. Malay, Chinese, and Indian adults. Age 61–95 years. 25 lived alone.	Six themes were identified: spirituality, physical health and function, peace of mind, financial independence, family, and the living environment. Participants reported that good physical health was an important resource that facilitated commitment to their spiritual activities. Participants wished for a “peaceful life” and experienced this by deepening their spirituality. Other ingredients for a peaceful life were financial independence, living in a place they love, and having family members who live in harmony. In this community where religious affiliation is a tradition, spirituality can be fundamental for healthy ageing, and its inclusion in eldercare policy is imperative.

(9) Tsai and Tsai (2007) TAIWAN [43]	Interviews. Conventional content analysis.	*N* = 9 (3 women, 6 men) All lived alone Age 65–90. 90% of the sample had a low level of dependence on help with activities of daily living.	The elderly persons' internal resources included self-perception of health status, preventive coping strategies, flexible coping ability, and being resigned to their situation. Their external resources were both human and environmental. Based on their lived experience, they appraised the usefulness of both internal and external resources before deciding whether to seek help from the latter.

(10) van Maanen (2006) CANADA [42]	Explorative design. Individual interviews. Comparative analysis.	*N* = 70 One group of healthy, elderly, community dwelling, white, American-born persons. One group of community dwelling British-born ill-healthy participants. Age from 65–84 and over 85 years. The British-born group suffered from different chronic diseases.	Self-defined healthy American-born and ill-healthy British-born elderly persons demonstrated that the perception of health is determined by more dimensions than the absence of disease and illness. The older the person, the more emphasis was placed on health as a state of mind, even with a gradually failing body. It was evident that these respondents, especially the ill-healthy elderly, challenged health providers' current beliefs about health and illness.

**Table 2:** Main themes, themes and sub-themes that emerged in the qualitative review of health and ill health among old persons and need of community health care.

Reconciliation with how life has become	Desire to restore loss of identity and self-worth in spite of disability
*Creating meaning and experience of worthiness in health care and society *	*A mental struggle to regain independency with the help of caregivers *

Maintaining balance, stability and adjustment	Loss of self-worth related to the changing body

The meaning of being responsible for others and society	

Togetherness	
